# Petunidin alleviates diabetic nephropathy injury via the inhibition of oxidative stress and ferroptosis through the Keap1/mitoNQO1 pathway

**DOI:** 10.3389/fcell.2025.1651382

**Published:** 2025-10-16

**Authors:** Yuli Qiu, Chao Chen, Xinyan Li, Yiling Chang, Xiaoqin Zou, Xiaopei Yan, Wenjun Mao, Gang Wu, Su Li, Yuqiong Chen

**Affiliations:** ^1^ Department of Nephrology, The Affiliated Suzhou Hospital of Nanjing Medical University, Suzhou Municipal Hospital, Gusu School, Nanjing Medical University, Suzhou, China; ^2^ Department of Cardiology, The Affiliated Suzhou Hospital of Nanjing Medical University, Suzhou Municipal Hospital, Gusu School, Nanjing Medical University, Taizhou, China; ^3^ National Cancer Center/National Clinical Research Center for Cancer/Cancer Hospital, Chinese Academy of Medical Sciences and Peking Union Medical College, Beijing, China; ^4^ The Affiliated Taizhou People’s Hospital of Nanjing Medical University, Jiangsu, China; ^5^ Department of Respiratory Medicine, The Affiliated Suzhou Hospital of Nanjing Medical University, Suzhou Municipal Hospital, Gusu School, Nanjing Medical University, Taizhou, China; ^6^ Department of Cardiology, Zhongshan Hospital, Fudan University, Shanghai, China

**Keywords:** diabetic nephropathy, oxidative stress, ferroptosis, mitochondria, petunidin, Keap1, NQO1

## Abstract

**Background:**

Diabetic nephropathy (DN) is one of the most serious complications of diabetes and the leading cause of end-stage renal disease worldwide. The pathogenesis of DN is complex, and oxidative stress and ferroptosis play key roles. Petunidin (PET) is a member of the anthocyanin family and has strong antioxidant activity. However, there are no relevant studies on the use of PET to improve diabetic nephropathy. The aim of this study was to investigate the protective mechanism of PET in diabetic nephropathy.

**Methods:**

In the animal experiments, db/m and db/db mice were treated with PET for 8 weeks. Renal function, urinary albumin/urinary creatinine ratio (ACR) and renal tissue section staining were used to observe renal pathological injury. For the cell experiments, normal renal cortex proximal convoluted tubule epithelial cells (HK-2 cells) were selected for further verification, and ADV-mediated Keap1 and mitoNQO1 overexpression models were constructed. Western blotting, immunofluorescence and TUNEL staining were used to detect oxidative stress- and ferroptosis pathway-related indicators.

**Results:**

Keap1 expression in the kidneys of db/db mice was significantly increased, along with reduced mitochondrial translocation of NQO1, while PET reversed this trend to decrease oxidative stress and inhibit ferroptosis. Further experiments confirmed that after overexpression of Keap1, the protective effect of PET in high glucose-induced HK2 cells disappeared, whereas overexpression of mitoNQO1 reduced oxidative stress and ferroptosis in a mitochondria-dependent way.

## 1 Introduction

Diabetes is a major public health challenge in both developed and developing countries. It is estimated that by 2030, there will be 643 million people with diabetes (Bibi Sadeer et al., 2020; Chen et al., 2011). Diabetic nephropathy is a common long-term complication of type 1 and type 2 diabetic nephropathy, and up to 35% of diabetic patients develop kidney disease, which is the main cause of chronic kidney disease (CKD) and end-stage renal disease (ESRD) requiring dialysis or transplantation worldwide. The pathogenesis of DN includes inflammation, oxidative stress, hemodynamic abnormalities, glucose and lipid metabolism disorders, genes, and fibrosis, and especially oxidative stress, which plays an important role (Chen et al., 2020; Kato and Natarajan, 2019). Recent molecular and cellular studies continue to explore new areas of diabetic complication pathogenesis, including genetic and epigenetic modifications, podocyte autophagy, and mitochondrial dysfunction, providing more possible directions for the treatment of DN (Chen et al., 2023; Lin et al., 2018).

The Keap1/Nrf2 signaling pathway has been shown to regulate antioxidant proteases, clear ROS, and maintain intracellular redox homeostasis (Sykiotis, 2021). Keap1 is a major intracellular regulator of Nrf2. Under homeostatic conditions, Nrf2 binds to its endogenous inhibitor Keap1, thus maintaining the inactivation state (Bellezza et al., 2010; Velichkova and Hasson, 2005). Oxidative stress causes Nrf2 to separate from Keap1 and transfer to the nucleus to activate the transcription of antioxidant response elements (AREs), increasing the expression of antioxidant and metabolic genes (Bellezza et al., 2018). NQO1, a target gene of Nrf2, has the enzymatic function of catalyzing and reducing a variety of compounds, including quinones, nitroaromatic compounds, imidazole, and iron ions (Pey et al., 2019; Ross and Siegel, 2004), and plays a role in ferroptosis. Zhan et al. reported that Plumbagin could target NQO1/GPX4-mediated ferroptosis and inhibit the growth of glioma *in vivo* and *in vitro* (Zhan et al., 2022). In addition, some studies have shown that Tanshinone helps NQO1 detoxify lipid peroxy radicals and inhibits ferroptosis both *in vitro* and *in vivo* (Wang et al., 2023).

Ferroptosis is a newly discovered form of programmed cell death characterized by iron-dependent lipid peroxidation-induced cell death (Chen et al., 2023; Chen Z. et al., 2024; Yin et al., 2025), which manifests mainly as reduced mitochondrial volume and mitochondrial ridge and increased mitochondrial membrane density (Lei et al., 2019). Biochemically, ferroptosis mainly manifests as decreased glutathione peroxidase-4 (GPX4) expression and activity, depletion of intracellular glutathione, and increased ROS levels. Iron accumulation, glutathione depletion, and lipid peroxidation are indispensable and occur simultaneously during ferroptosis (Liu et al., 2020). Ferroptosis is accompanied by the production of excess lipid ROS, which can lead to oxidative stress, and enriched kidney mitochondria are more vulnerable to oxidative stress damage. The traditional pathogenesis of DN involves oxidative stress, suggesting that ferroptosis may be related to DN. Yue Wang et al. first reported that ferroptosis is involved in DN by exploring the role of ferroptosis in the progression of DN via *in vivo* and *in vitro* experiments and reported that the expression of the ferroptosis-related protein GPX4 was decreased, ACSL4 expression was increased, and lipid peroxide products and iron content were also increased in mouse models of DN (Wang et al., 2020).

Anthocyanins are the main components of water-soluble pigments in plants and are flavonoids. Anthocyanins have been found to be beneficial in the prevention and treatment of diabetes and its complications because of their ability to reduce hyperglycemia, insulin resistance, reactive substances, and proinflammatory cytokines. In addition, they have been found to be involved in gluconeogenic inhibition, as well as α-amylase and α-glucosidase activity (Kato and Natarajan, 2019). Compared with other flavonoids, anthocyanins have greater antioxidant activity, and their mechanism of action may involve the mitochondrial pathway (Liobikas et al., 2016). Petunidin (PET) is an anthocyanin found in many red berries that inhibits the level of MDA and increases the contents of SOD and GSH to reduce oxidative stress (Cai et al., 2020). Paulina Strugała et al. first reported the antidiabetic and antioxidant effects of PET on STZ-induced diabetic rats (Strugała et al., 2019). However, the role of PET in diabetic nephropathy remains unknown. Therefore, this study aimed to explore the potential role of PET in DN both *in vivo* and *in vitro* and explored its potential signaling pathways in an attempt to elucidate the exact role of mitochondrial oxidative stress and ferroptosis.

## 2 Materials and methods

### 2.1 Animal experiments

8-weeks-old male C57BLKS/J db/db mice and control db/m mice were purchased from Gem Pharmatech Co., Ltd. (Nanjing, China). They are type 2 diabetes model mice due to a spontaneous mutation and have obvious characteristics of type 2 diabetes (Li et al., 2023). The animal experiment protocol was approved by the Institutional Animal Use and Care Committee of Nanjing Medical University (Ethical No. IACUC-2204022) and was conducted in strict accordance with the Guidelines for the Use and Management of Laboratory Animals issued by the National Institutes of Health of China. All the mice were kept in an SPF room at a temperature of 22 °C ± 2 °C, a relative humidity of 50% ± 5%, a 12 h light-dark cycle, and provided food and water freely. After 2 weeks of adaptation, unilateral nephrectomy was performed on the mice. The mice were randomly divided into four groups (n = 6): the db/m group, db/m + PET group, db/db group and db/db + PET group. Eight weeks after the surgery, the PET treatment groups were given 10 mg/kg/d PET by gavage for 8 weeks according to previous studies (Nagaoka et al., 2019). Then, all the mice were anesthetized via intraperitoneal injection of pentobarbital sodium (30 mg/kg) and sacrificed via cervical dislocation. Blood samples were collected and stored at −80 °C for later use. The kidney tissues were partially fixed in 4% paraformaldehyde (PFA) for histological examination.

### 2.2 Cell culture and treatment

The human proximal renal tubule cell line (HK2) was supplemented with 10% bovine serum (ServiceBio, Wuhan, China), 100 U/mL penicillin and 100 μg/mL streptomycin (Gibco, China) and was cultured in 5.5 mmol/L glucose DMEM/F-12 (BasalMedia, Shanghai, China) at 37 °C, 95% humidity and 5% CO_2_. HG intervention: HK2 cells received culture medium supplemented with 30 mmol/L glucose for 48 h. PET treatment: Petunidin chloride (purity ≥99%) was obtained from MCE (United States). PET was dissolved in DMSO, and then added to the cells for 48 h at different concentrations (25, 50, or 100 μM).

### 2.3 Biochemical detection

Body weight and glucose were assessed every 4 weeks. The mice were placed in a metabolic cage for 24 h, and urine was collected at week 0 and week 26. Mouse urine samples were centrifuged at 4 °C for 15 min, and the supernatant was collected. The concentrations of urinary albumin and creatinine were determined via a mouse urine protein ELISA kit (Shanghai Viao, China). Then, the urinary albumin/urinary creatinine ratio (ACR) was calculated. Mouse serum creatinine and blood urea nitrogen (BUN) were tested by ELISA kit (Shanghai Viao, China). A total of 10 mg of kidney tissue from each sample was ground to obtain the supernatant. The supernatant was centrifuged at 4 °C for 15 min, and the results were measured with an ELISA kit (Nanjing Jiancheng Bioengineering Institute, Nanjing, Jiangsu, China) according to the manufacturer’s instructions.

### 2.4 Immunohistochemistry and immunofluorescence staining

The kidney tissues were fixed with 4% paraformaldehyde (PFA), embedded in paraffin, and cut into 4 μm thick slices. The kidney sections were stained with hematoxylin-eosin (HE), Schiff periodate (PAS), Masson and Sirius Red.

For immunofluorescence staining, the frozen sections were infiltrated with 0.3% Triton X-100 for 10 min, blocked with 3% BSA for 1 h, and incubated with the primary antibody at 4 °C overnight. On the second day, after the samples were washed with PBS, they were incubated with a fluorescent secondary antibody (1:500) at room temperature in the dark for 1 h. DAPI (1:500) was applied 8 min before fluorescence microscopy. The samples were observed via confocal microscopy, and the fluorescence intensity was analyzed via ImageJ software. The immunofluorescence procedure for cells was similar to that for kidney tissues. The primary antibodies used for immunofluorescence are shown in Supplementary Table S1.

### 2.5 Separation of cytoplasmic and mitochondrial fractions

Fresh kidney tissues and cultured HK2 cells were washed with PBS, mitochondria separation reagent (Beyotime, Shanghai, China) was added, they were homogenized in an ice bath approximately 10 times, and centrifuged at 4 °C for 5 min. The supernatant was transferred to another centrifuge tube and centrifuged at 11,000 × g at 4 °C for 10 min. The supernatant contained the isolated cytoplasm, and the precipitate contained the isolated mitochondria.

### 2.6 Measurement of oxidative damage biomarkers

Protein samples were extracted from mouse kidney tissues, HK2 cells, or isolated mitochondria via lysis and centrifugation. In accordance with the manufacturer’s instructions, MDA (Nanjing Jiancheng Bioengineering Institute, Nanjing, Jiangsu, China) test kits were used to measure the contents of MDA. The glutathione (GSH) and catalase (CAT) activities were determined via GSH and CAT assay kits (Beyotime, China). Superoxide dismutase (SOD) and manganese-SOD (Mn-SOD) activities were measured via Cu/Zn-SOD and Mn-SOD assay kits with WST-8 (Beyotime, China) (Chen et al., 2025b).

### 2.7 Measurement of 12-HETE, 15-HETE, and 4-HNE levels

To determine 12-hydroxyeicosatetraenoic acid (12-HETE) and 15-HETE levels, 12-HETE and 15-HETE ELISA kits (Abcam, United States) were used according to the product instructions. To detect 4-hydroxynonenal (4-HNE), the OxiSelectTM 4-HNE Assay Kit (Cell Biolabs Inc., United States) was utilized following the kit’s instruction manual.

### 2.8 Iron content detection

Total iron content was measured with an iron assay kit (Abcam) according to the product instructions. Briefly, mouse kidney tissue or HK2 cells in iron assay lysis buffer were rapidly homogenized and centrifuged at 16,000 × g at 4 °C for 10 min. The supernatant was then incubated with an iron reducer at 25 °C for 30 min, followed by incubation with iron probe buffer for 60 min at 25 °C in the dark. A microplate reader (Molecular Devices) was used to read the absorbance at 593 nm.

### 2.9 Lipid peroxidation analysis

The lipid peroxidation (LPO) content was measured via a lipid peroxidation assay kit according to the manufacturer’s instructions (Nanjing Jiancheng Bio, China). In brief, the chromogen reagent was combined with lipid peroxide and incubated at 45 °C for 60 min to stabilize the chromophore. Readings were subsequently obtained via a microplate reader at 586 nm. The lactate dehydrogenase (LDH) release assay was conducted using an LDH cytotoxicity assay kit (MedChemExpress, Monmouth Junction, China).

### 2.10 Western blotting

The protein from kidney tissues and HK2 cells was extracted from the lysate, and the concentration was determined via the bicinchoninic acid (BCA) method. The protein extracts were separated and electrophoresed on a 10% polyacrylamide gel (SDS‒PAGE) at voltages of 80 V, 30 min and 100 V for 90 min. Then, the gel was transferred to a PVDF membrane. The membrane was blocked with fast block blocking buffer for 15 min. Primary antibodies were added to the membrane overnight at 4 °C. The membrane was subsequently washed with TBS-T and incubated with secondary antibodies for an hour at RT. The bands were displayed on a Tanon-5200 Multi. β-actin was used as an internal reference. The primary antibodies used for Western blotting are shown in Supplementary Table S1.

### 2.11 TUNEL analysis

Apoptosis was measured with a TUNEL apoptosis assay kit. Kidney tissues were fixed with 4% PFA and infiltrated with 0.1% Triton X-100. Then, the samples were incubated with 50 μL of biotin-dUTP buffer at 37 °C for 1 h, and the positive samples were observed via fluorescence microscopy (Olympus, Tokyo, Japan).

### 2.12 Cell viability assay

Cell viability was measured using a CCK-8 assay (Topscience, Shanghai, China) according to the manufacturer’s instructions. HK2 cells were cultured in 96-well plates, incubated at 37 °C for 12 h, and then cultured in 25, 50, or 100 μM PET containing 30 mM glucose for 48 h. 100 μL of CCK-8 working solution were added to each well. A microplate reader was used to measure the absorbance at 450 nm.

### 2.13 Detection of ROS and mitochondrial membrane potential

In accordance with the manufacturer’s instructions, the ROS levels in renal tissues and HK2 cells were measured using a dihydroethyl (DHE, Sigma) probe and a 2′,7′-dichlorodihydrofluorescein (DCFH-DA) fluorescent probe (Beyotime, Shanghai, China) and then observed under an inverted microscope.

The mitochondrial membrane potential was detected with a JC-1 fluorescent probe (Beyotime, Shanghai, China). One milliliter of JC-1 dye solution was added, the mixture was incubated at 37 °C for 20 min, and then the mixture was washed with JC-1 dyeing buffer twice. An inverted microscope was used for image acquisition.

### 2.14 Cellular thermal shift assay (CETSA)

HK2 cells were collected and freeze-thawed under liquid nitrogen, and cell lysates were diluted with PBS and divided into two aliquots, with one aliquot being added to PET and the other being added to DMSO as control. After incubation at 37 °C for 1 h, all lysates were equally divided into nine PCR tubes, and each tube of cells was heated separately at different temperatures (37 °C, 40 °C,43 °C, 49 °C, 52 °C, 55 °C, 58 °C, 61 °C, and 64 °C) for 3 min. After that, samples were centrifuged at 4 °C, and the soluble fractions were subjected to Western blot analysis (Chen et al., 2025a; Fu et al., 2025).

### 2.15 Statistical analysis

All data are expressed as the mean ± standard error of the mean (SEM). Statistical analysis was performed with GraphPad Prism 9 software (San Diego, CA). Student’s t test, one-way ANOVA followed by Tukey’s test, and two-way ANOVA followed by Tukey’s test were performed for comparisons. P values <0.05 were considered significantly different.

## 3 Results

### 3.1 Effects of PET on physicochemical properties and renal pathological changes in db/db mice

**FIGURE 1:**
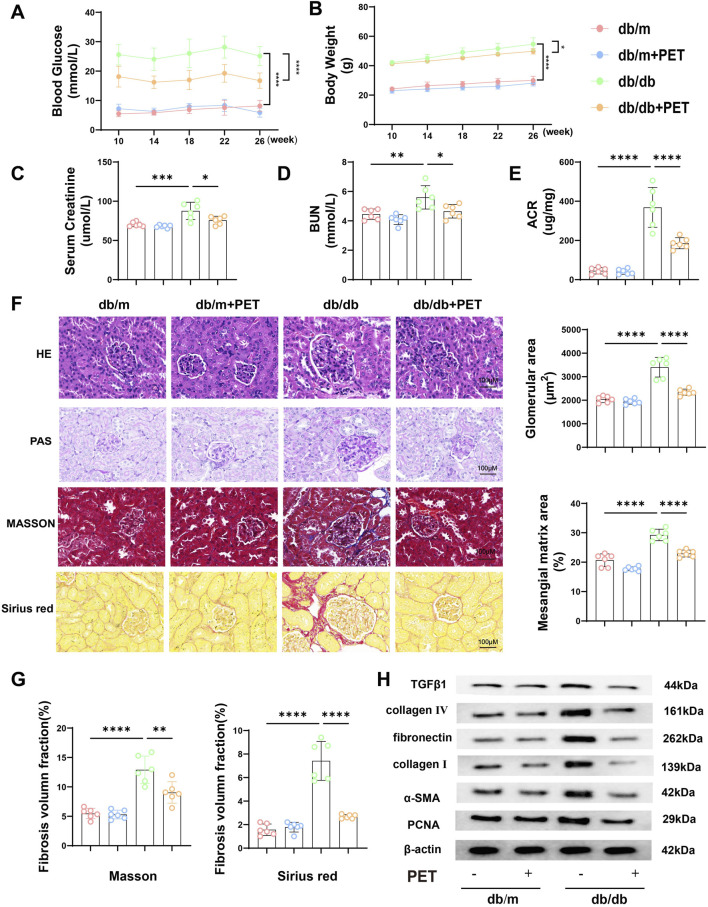
Effects of PET on biological properties and renal pathological changes in db/db mice. **(A)** Changes in blood glucose. **(B)** Changes in body weight. **(C)** Serum creatinine level. **(D)** BUN levels. **(E)** Urine ACR level. **(F)** Representative images of HE, PAS, Masson and Sirius Red staining of kidney tissues. Scale bars = 100 μm. **(G)** Fibrosis volume fraction in Masson and Sirius Red staining of kidney tissues. **(H)** Western blot analysis of the levels of fibrosis-related proteins in kidney tissues. The results are presented as the mean ± SEM. *P < 0.05. **P < 0.01. ***P < 0.001. ****P < 0.0001. Six biological replicates were included in each experiment.

Whether PET can alleviate diabetic kidney damage is not known. For this purpose, we treated db/db mice with PET by gavage. Compared with those of the db/m group, the blood glucose and body weight of the db/db group were significantly increased, whereas those of the PET group decreased (Figures 1A,B). The results suggested PET has an antidiabetic effect, which is consistent with previous studies (Strugała et al., 2019). Pet alleviated the indicators of diabetic kidney injury, including serum creatinine, BUN and urinary ACR in db/db mice (Figures 1C–E). The effects of PET on the morphological changes in db/db mice were observed via HE, PAS, Masson and Sirius Red staining. HE and PAS staining revealed obvious pathological changes in the kidney tissues of db/db mice, including glomerular hypertrophy, mesangial matrix dilatation, tubular lumen dilatation and glycogen deposition, which were alleviated by PET treatment (Figure 1F). The results from Masson and Sirius red staining and Western blot analysis of fibrosis-related proteins revealed that PET treatment mitigated renal fibrosis (Figures 1F–H). Thus, PET attenuated db/db mouse kidney injury.

### 3.2 Pet alleviated renal and mitochondrial oxidative stress in db/db mice

**FIGURE 2:**
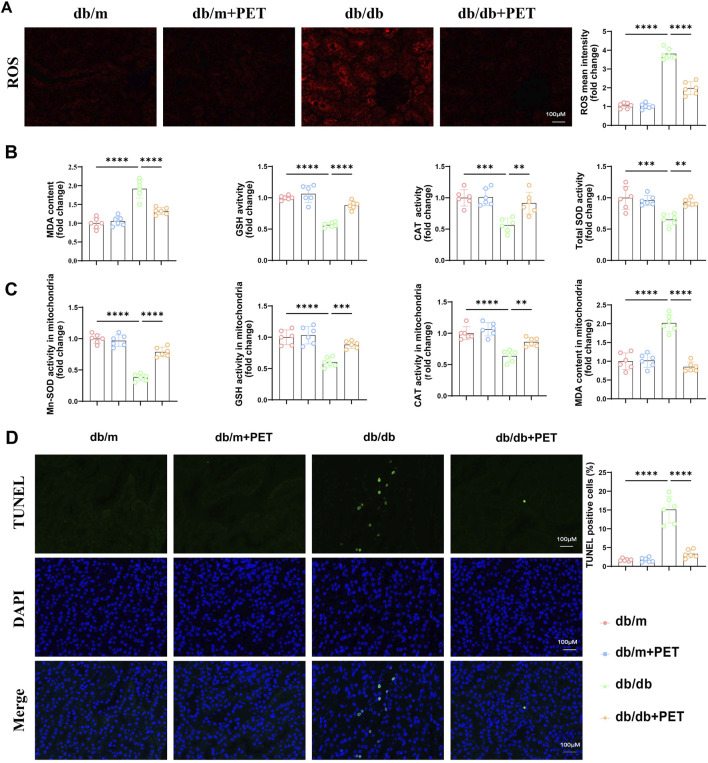
Pet alleviated renal and mitochondrial oxidative stress in db/db mice. **(A)** Representative immunofluorescence images and quantitative analysis of ROS levels in kidney tissues. Scale bars = 100 μm. **(B)** The contents of MDA and the activities of GSH, CAT, and SOD in kidney tissues. **(C)** The activities of Mn-SOD, GSH, CAT, and content of MDA in mitochondria isolated from kidney tissues. **(D)** Representative images and quantitative analysis of TUNEL-stained kidney tissues. Scale bars = 100 μm. The data are expressed as the mean ± SEM. *P < 0.05. **P < 0.01. ***P < 0.001. ****P < 0.0001. Six biological replicates were included in each experiment.

Given that PET has previously been shown to have antioxidative stress effects, we investigated whether PET treatment could mitigate oxidative stress in db/db mice. PET treatment inhibited the increase in renal ROS levels in db/db mice (Figure 2A). Compared with those in the db/m group, the MDA levels were significantly increased, and the GSH, CAT and total SOD activities were significantly decreased in the db/db group (Figure 2B). In contrast, PET treatment reversed these oxidative stress injuries in db/db mice (Figure 2B).

Mn-SOD (SOD2) is an essential mitochondrial antioxidant regulatory enzyme that plays an important role in regulating mitochondrial homeostasis (Chen et al., 2020). We found that PET could increase the activity of Mn-SOD in kidney tissue (Figure 2C) and we further evaluated markers of mitochondrial oxidative stress in db/db mouse kidney tissue. The MDA content increased, and the GSH and CAT activities decreased in the mitochondria of db/db mice, which was alleviated by PET treatment (Figure 2C). TUNEL staining revealed that PET reduced apoptosis in diabetic renal tissue (Figure 2D). These results indicated that PET treatment could alleviate oxidative stress in renal tissue and mitochondria.

### 3.3 PET reduced oxidative stress injury by targeting Keap1 and NQO1 for mitochondrial translocation

**FIGURE 3:**
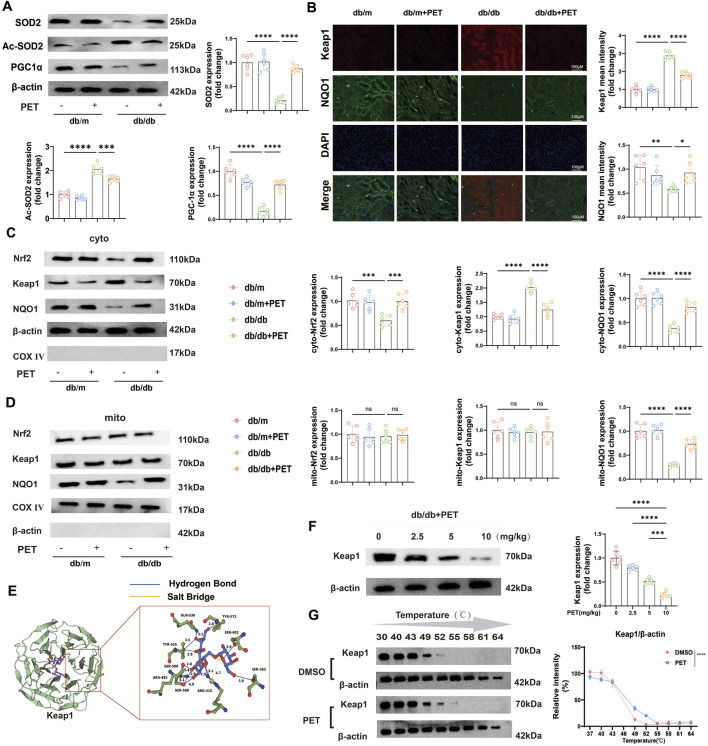
PET reduced oxidative stress injury by targeting Keap1 and NQO1 for mitochondrial translocation. **(A)** Western blot quantitative analysis of SOD2, Ac-SOD2, and PCG-1α protein expression in kidney tissues. **(B)** Representative immunofluorescence images and quantitative analysis of the oxidative stress-related proteins Keap1 and NQO1 in kidney tissues. Scale bars = 100 μm. **(C,D)** Western blot quantitative analysis of the levels of oxidative stress-related proteins in kidney tissues and mitochondria. **(E)** Molecular docking assay between PET and Keap1. **(F)** The link between PET dose and Keap1 protein expression was detected by Western blot analysis and quantified. **(G)** Cellular thermal shift assay detecting the thermal stability of Keap1 after PET treatment in HK2. The data are expressed as the mean ± SEM. *P < 0.05. **P < 0.01. ***P < 0.001. ns, not significant. Six biological replicates were included in each experiment.

Previously, we confirmed that SOD2 levels were reduced in mitochondria in db/db mice and that PET reversed this trend. SOD2 inactivation is associated with its acetylation (Ac), and PGC-1α expression is a key indicator for evaluating mitochondrial biosynthesis. Western blot analysis revealed decreased levels of SOD2 and PGC-1α and increased levels of Ac-SOD2 in db/db mice, while PET treatment reversed these trends (Figure 3A), which suggested that PET could improve mitochondrial function in diabetes.

To further evaluate the specific molecular mechanism under PET treatment, we detected the expression levels of mitochondrial oxidative stress-related proteins. The Keap1/Nrf2 pathway plays an important role in the regulation of oxidative stress. The results of renal tissue immunofluorescence revealed that Keap1 expression increased and Nrf2 expression decreased in db/db mice, whereas PET treatment reversed these trends (Figure 3B). In view of the significant improvement in mitochondrial function by PET, the distributions of proteins related to the Keap1/Nrf2 pathway in the cytoplasm and mitochondria were further clarified. The results revealed that only the mitochondrial distribution of NQO1 were significantly decreased (Figures 3C,D). However, PET treatment significantly facilitated the mitochondrial translocation of NQO1 (Figures 3C,D). Moreover, among the proteins associated with oxidative stress, significant changes in Keap1 expression were observed (Figure 3C). Therefore, it was reasonable to hypothesize that PET might reduce oxidative stress damage by targeting Keap1 and NQO1 mitochondrial translocation (Figures 3C,D).

Given the significant changes in Keap1 protein levels, we speculated that PET might influence Keap1 protein expression and predicted the effect of PET on the Keap1 protein via molecular docking methods. The results revealed that 8 hydrogen bonds and 2 salt bonds formed between PET and Keap1, with a predicted docking score of −11.48. This finding suggested a possible direct interaction between PET and Keap1 (Figure 3E). To study the relationship between PET and Keap1, db/db mice were treated with different concentrations of PET (2.5, 5, or 10 mg/kg), and the results revealed that the Keap1 protein expression level was negatively correlated with the PET concentration (Figure 3F). Furthermore, the cellular thermal shift assay (CETSA) was used to demonstrate the biological interaction between Keap1 and PET, which showed that PET incubation led to the stabilization of Keap1 (Figure 3G). These data suggest that Keap1 might be a potential target for PET to ameliorate oxidative stress.

### 3.4 PET inhibited ferroptosis in db/db mice

**FIGURE 4:**
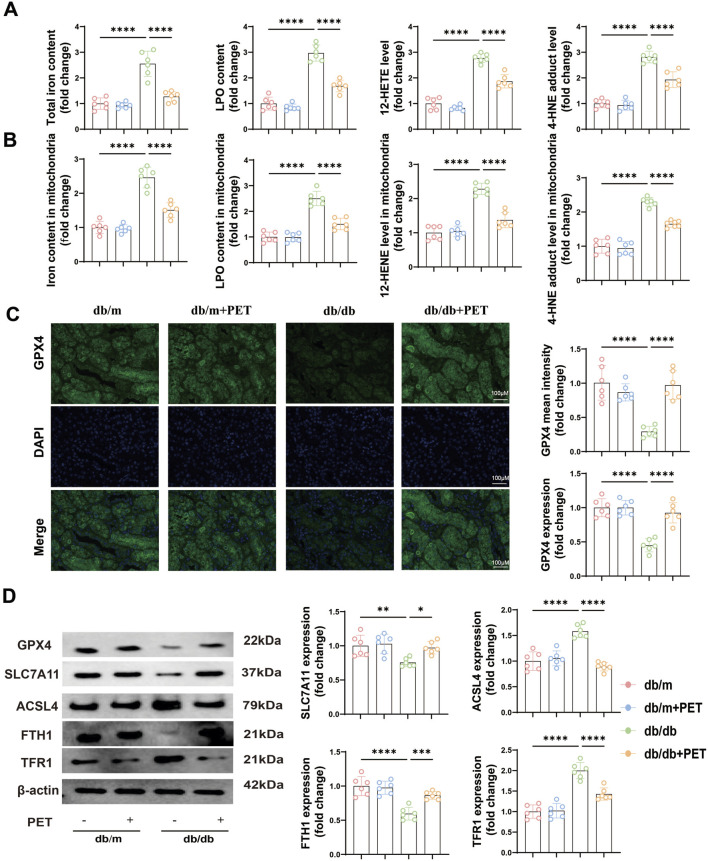
PET inhibited ferroptosis in db/db mice. **(A,B)** The contents of total iron and LPO and the levels of 12-HETE and 4-HNE in kidney tissues and mitochondria. **(C)** Representative images and quantitative analysis of GPX4 in kidney tissues by immunofluorescence. Scale bars = 100 μm. **(D)** Western blot analysis of GPX4, SLC7A11, ACSL4, FTH1 and TFR1 protein expression in kidney tissues. The data are expressed as the mean ± SEM. *P < 0.05. **P < 0.01. ***P < 0.001. ****P < 0.0001. Six biological replicates were included in each experiment.

NQO1 is a target gene of NRF2 and plays a role in ferroptosis (Zuo et al., 2023). We next explored whether the oxidative stress injury caused by diabetes was associated with ferroptosis. Ferroptosis is related to iron overload, lipid peroxidation and weakened antioxidant capacity. The results revealed that the levels of total iron, LPO, 12-HETE and 4-HNE in the cytoplasm and mitochondria were significantly increased in db/db mice, whereas treatment with PET resulted in the opposite trend (Figures 4A,B). GPX4, SLC7A11, ACSL4, FTH1, and TFR1 are the key indicators of ferroptosis. Immunofluorescence staining and Western blot analysis were used to assess the levels of ferroptosis-associated proteins. Immunofluorescence staining revealed reduced GPX4 expression in db/db mice, and PET treatment increased GPX4 expression (Figure 4C). Furthermore, Western blot analysis revealed that the expression of GPX4, SLC7A11, and FTH1 decreased in db/db mice and that the expression of ACSL4 and TFR1 increased, the effects of which were reversed by PET treatment (Figure 4D). These findings suggested that PET could improve ferroptosis in db/db mice.

### 3.5 Pet alleviated HG-induced oxidative stress in HK2 cells by inhibiting Keap1 expression

**FIGURE 5:**
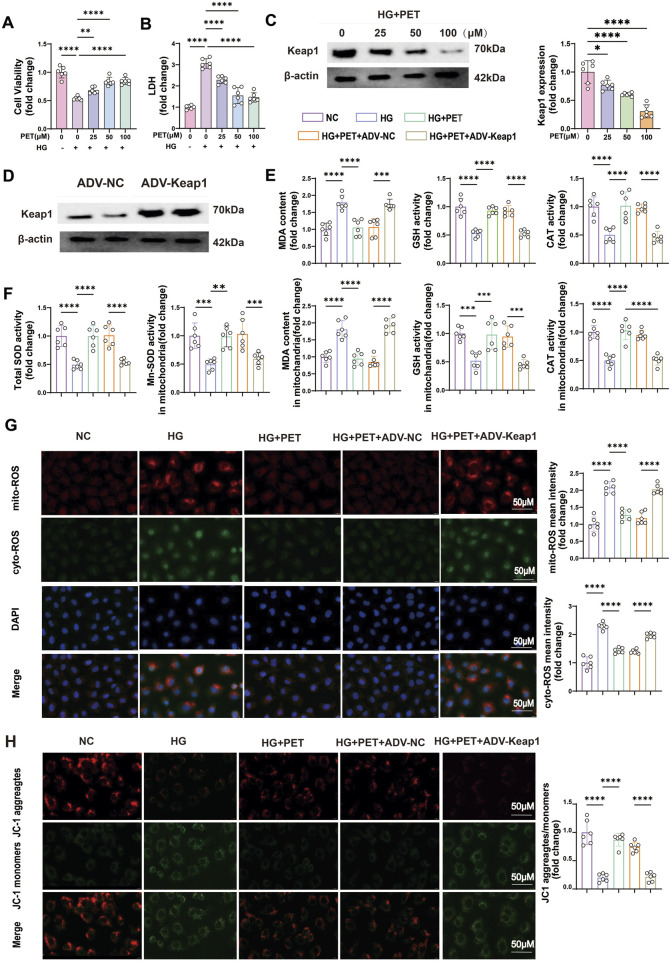
Pet alleviated HG-induced oxidative stress in HK2 cells by inhibiting Keap1 expression. **(A,B)** Cell viability and LDH content were detected in HG-induced HK2 cells treated with increasing PET doses. **(C)** Keap1 expression was detected with increasing PET dose via Western blot analysis in HG-induced HK2 cells. **(D)** Western blot verification of Keap1 overexpression in HK2 cells. **(E,F)** Quantitative analysis of cellular and mitochondrial oxidative stress markers. **(G)** Representative images and quantitative analysis of mitochondrial (red) and cellular (green) ROS. Scale bar = 50 μm. **(H)** Representative images and quantitative analysis of the mitochondrial membrane potential were obtained via JC-1 staining. Scale bar = 50 μm. The data are expressed as the mean ± SEM. *P < 0.05. **P < 0.01. ***P < 0.001. ****P < 0.0001. Six biological replicates were included in each experiment.

Furthermore, we confirmed the protective effect of PET on oxidative stress in HK2 cells *in vitro*. HK2 cells were cultured in medium supplemented with 30 mM glucose and treated with different PET concentrations (0, 25, 50, and 100 μM). PET treatment increased cell viability, reduced LDH release, and suppressed Keap1 expression in a dose-dependent manner (Figures 5A–C). The benefits of PET reached peak at 50 μM, therefore, 50 μM was chosen as the dose for subsequent *in vitro* experiments.

To further investigate the role of Keap1 in oxidative stress, we overexpressed the Keap1 gene in HK2 cells via viral transfection (Figure 5D). In accordance with the animal experiments, PET treatment alleviated cellular and mitochondrial oxidative stress injuries in HK2 cells (Figures 5E–G). Additionally, Pet alleviated mitochondrial injury as evidenced by improved mitochondrial membrane potential (Figure 5H). However, after the overexpression of Keap1, the beneficial effects of PET were compromised (Figures 5E–H). The above experimental results indicated that Pet alleviated HG-induced oxidative stress in HK2 cells by inhibiting Keap1.

### 3.6 PET stabilized mitochondrial function and reduced ferroptosis in HK2 cells via Keap1 downregulation

**FIGURE 6:**
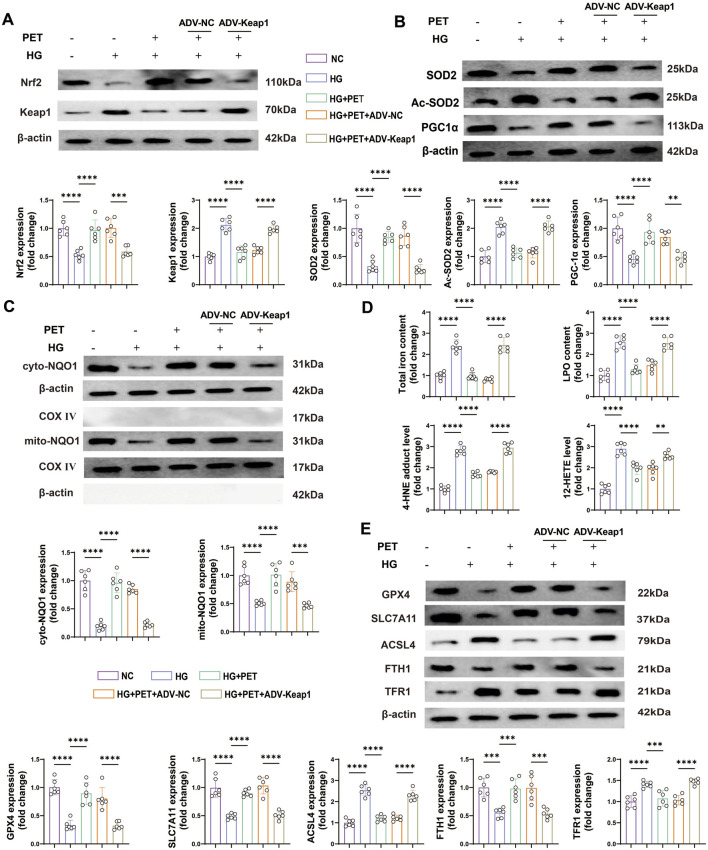
PET stabilized mitochondrial function and reduced ferroptosis in HK2 cells via Keap1 downregulation. **(A,B)** Representative Western blot analysis of oxidative stress and mitochondrial function-related proteins. **(C)** Representative Western blot analysis of NQO1 in the cytoplasm and mitochondria of HK2 cells. **(D)** The contents of total iron and LPO and the levels of 12-HETE and 4-HNE. **(E)** Western blot quantitative analysis of ferroptosis markers in HK2 cells. The data are expressed as the mean ± SEM. *P < 0.05. **P < 0.01. ***P < 0.001. ****P < 0.0001. Six biological replicates were included in each experiment.

To further demonstrate that PET affects oxidative stress and mitochondrial function by targeting Keap1, we examined the expression levels of a series of oxidative stress-related proteins. The results showed that PET treatment increased the protein expression levels of Nrf2 in HK2 cells induced by high glucose but decreased the protein expression level of Keap1 (Figure 6A). However, the overexpression of Keap1 almost completely blocked these PET-mediated changes (Figure 6A). PET increased the protein levels of SOD2 and PGC-1α and alleviated HG-induced acetylation of SOD2, which was abolished by Keap1 overexpression (Figure 6B). These data further suggested that PET improved oxidative stress damage and improved mitochondrial function by inhibiting Keap1.

Animal experiments revealed that PET inhibited Keap1 expression and promoted the mitochondrial translocation of NQO1 in the kidney tissue of db/db mice. Therefore, in subsequent experiments, we further investigated the role of NQO1 in high glucose-stimulated HK2 cells and its relationship with Keap1. PET treatment significantly increased the expression level of NQO1, and promoted the translocation of NQO1 to mitochondria, the effects of which were obviously inhibited by Keap1 overexpression (Figure 6C), indicating that PET promoted NQO1 upregulation and mitochondrial translocation by inhibiting Keap1.

Similarly, we investigated the effects of PET on ferroptosis in HK2 cells. The total iron and LPO contents and the levels of 12-HETE and 4-HNE increased in the high-glucose intervention group, which was reversed by PET treatment (Figure 6D). In addition, Western blot analysis revealed that PET treatment effectively attenuated high glucose-induced ferroptosis, but was abolished by the overexpression of Keap1 (Figure 6E). Taken together, these results suggested that Pet alleviated high glucose-induced oxidative stress, impaired mitochondrial function and promoted ferroptosis by regulating Keap1 in HK2 cells.

### 3.7 PET attenuated HG-induced oxidative stress injury in HK-2 cells via the Keap1/mitoNQO1 pathway

**FIGURE 7:**
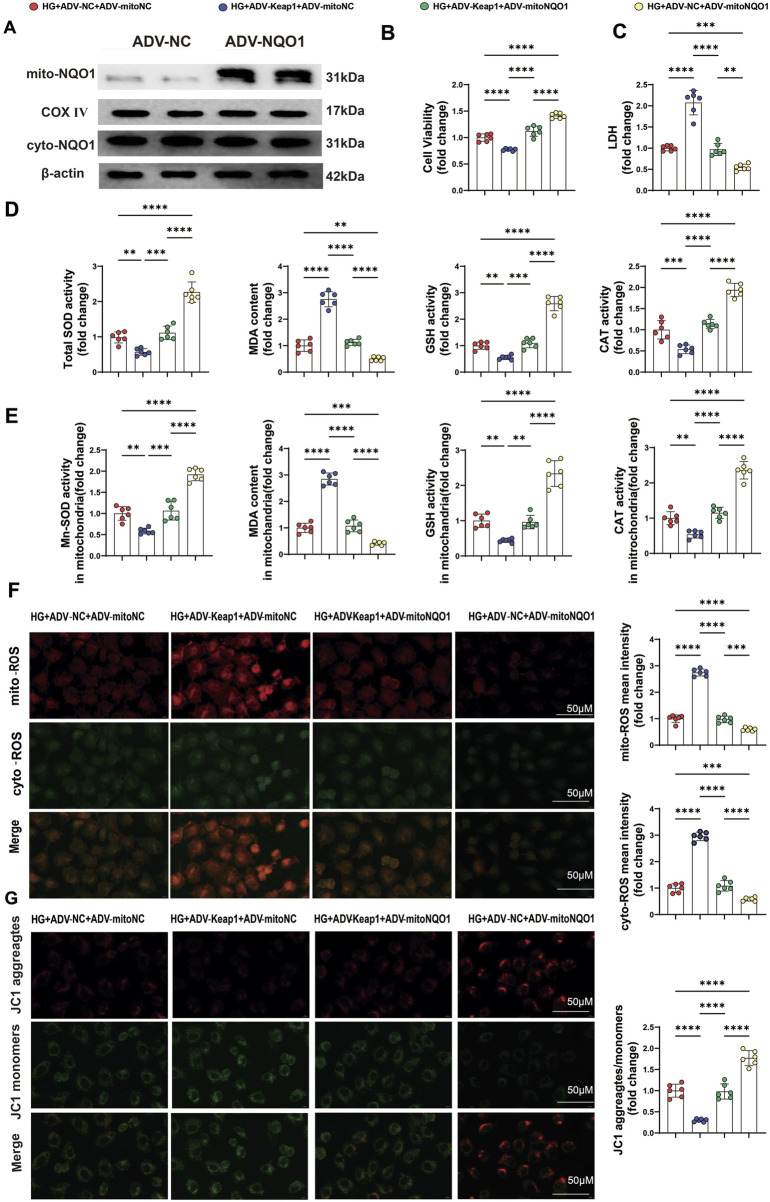
PET attenuated HG-induced oxidative stress injury in HK-2 cells via the Keap1/mitoNQO1 pathway. **(A)** mitoNQO1 overexpression transfection efficiency was determined by Western blot analysis. **(B,C)** Cell viability and LDH content were determined in HK2 cells. **(D,E)** Quantitative analysis of cellular and mitochondrial oxidative stress markers. **(F)** Representative images and quantitative analysis of mitochondrial (red) and cellular (green) ROS. Scale bar = 50 μm. **(G)** Representative images and quantitative analysis of the mitochondrial membrane potential were obtained via JC-1 staining. Scale bar = 50 μm. The data are expressed as the mean ± SEM. *P < 0.05. **P < 0.01. ***P < 0.001. ****P < 0.0001. Six biological replicates were included in each experiment.

To further confirm the role of mitochondrial NQO1 in alleviating oxidative stress, NQO1 with a mitochondrial translocation peptide (mitoNQO1) was overexpressed in HK2 cells (Figure 7A). Western blotting was used to detect the transfection efficiency and specificity of mitoNQO1 (Figure 7A). As previously shown, overexpression of Keap1 significantly inhibited cell viability and increased cell damage under high-glucose conditions, and these effects were eliminated by overexpression of mitoNQO1 (Figures 7B,C). In addition, when Keap1 was overexpressed, cellular and mitochondrial MDA and ROS contents increased, and cellular and mitochondrial GSH, CAT, total SOD and Mn-SOD activities and the mitochondrial membrane potential decreased (Figures 7D–G). However, these effects of Keap1 overexpression were largely eliminated in the case of mitoNQO1 overexpression (Figures 7D–G). These findings confirmed the key roles of the Keap1/mitoNQO1 pathway in PET-mediated renal protection against oxidative stress and mitochondrial function under high-glucose conditions.

### 3.8 PET attenuated HG-induced oxidative stress-related proteins and ferroptosis in HK-2 cells via the Keap1/mitoNQO1 pathway

**FIGURE 8:**
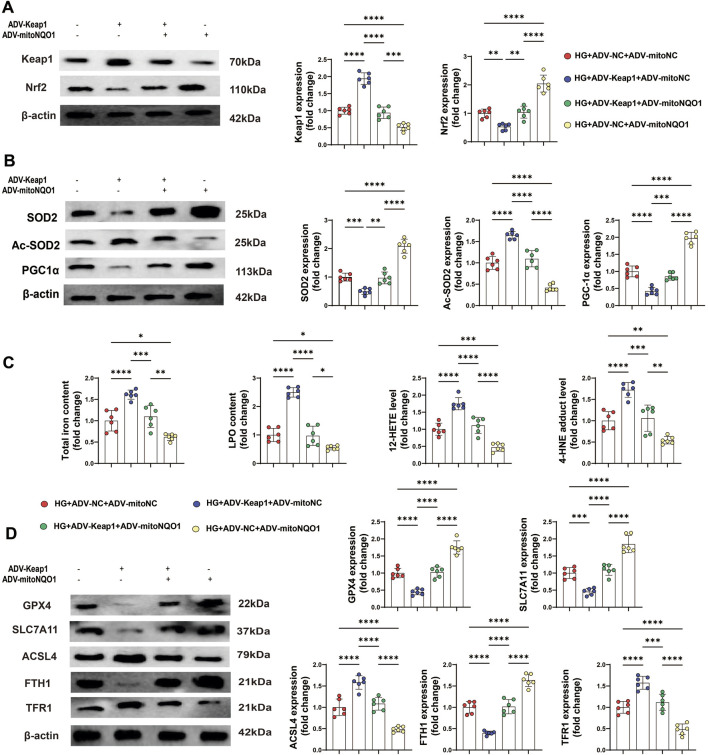
PET attenuated HG-induced oxidative stress-related protein expression and ferroptosis in HK-2 cells via the Keap1/mitoNQO1 pathway. **(A,B)** Western blot analysis of the levels of proteins related to oxidative stress and mitochondrial function in HK2 cells. **(C)** The content of total iron and LPO and the levels of 12-HETE and 4-HNE in HK2 cells. **(D)** Western blot analysis of ferroptosis markers. The data are expressed as the mean ± SEM. *P < 0.05. **P < 0.01. ***P < 0.001. ****P < 0.0001. Six biological replicates were included in each experiment.

To further study the influence of the Keap1/mitoNQO1 pathway on oxidative stress, Western blotting was also performed, and the results revealed that Keap1 overexpression suppressed Nrf2 expression under high glucose conditions, whereas mitoNQO1 overexpression promoted its expression (Figure 8A). The Western blotting results revealed decrease protein levels of SOD2 and PGC-1α, along with an increase in the protein level of Ac-SOD2 caused by Keap1 overexpression, whereas mitoNQO1 overexpression counteracted these harmful effects (Figure 8B).

In addition, overexpressing mitoNQO1 alleviated ferroptosis under Keap1 overexpression, primarily evidenced by reduced the levels of 12-HETE and 4-HNE, as well as the total iron and LPO contents (Figure 8C). Moreover, the expression of ferroptosis-related proteins could be improved by mitoNQO1 overexpression (Figure 8D). Together, these findings suggest a permissive role of the Keap1/mitoNQO1 pathway in PET-mediated renal protection against ferroptosis under high-glucose conditions.

## 4 Discussion

Our results suggested that PET inhibited Keap1 expression to upregulate the expression of NQO1 and promote mitochondrial translocation. This alleviated oxidative stress and ferroptosis both *in vivo* and *in vitro* and ultimately ameliorated diabetes-induced renal tubular epithelial cell injury and DN. Therefore, we propose that PET might be a promising therapeutic candidate and that the Keap1/mitoNQO1 pathway might represent a new therapeutic target for the treatment of DN.

Oxidative stress and mitochondrial dysfunction are important in the pathogenesis of DN (Jha et al., 2016). An excess of ROS is produced in DN (Eid et al., 2009). Ferroptosis is an iron-dependent form of programmed cell death driven by disturbances in iron metabolism and excess lipid peroxides, which are associated with oxidative stress (Chen Y. et al., 2024; Galaris et al., 2019; Mengstie et al., 2023). Previous studies have shown that reducing oxidative stress damage and ferroptosis could prevent podocyte damage (Zhang et al., 2021), alleviate renal tubule injury and delay renal fibrosis (Lo et al., 2022). Consistent with these studies, in the present study, we confirmed that in db/db mice and high glucose-induced HK2 cells, iron overload, increased ROS production, decreased mitochondrial membrane potential, increased oxidative stress-related indices, increased lipid peroxidation product MDA, and increased iron death-related indices, whereas PET reversed these changes to a certain extent and alleviated renal fibrosis. These results suggested that PET could delay the progression of DN by inhibiting oxidative stress and ferroptosis.

Keap1 is a cysteine-rich protein that acts as a redox damage sensor and is a major intracellular regulator of Nrf2 (Raghunath et al., 2018). The Keap1 protein harbors highly conserved and reactive cysteine residues that serve as electrophilic sensors responsive to both endogenous and exogenous reactive oxygen species. Three critical cysteine residues are identified in Keap1: Cys151 within the BTB domain, along with Cys273 and Cys288 in the IVR region. Notably, Cys151 functions as the primary sensing site for oxidative stress, where its modification disrupts the Keap1-Cul3 interaction and subsequently triggers Nrf2 dissociation from Keap1 (Yu and Xiao, 2021).Under physiological conditions, Keap1 captures and ubiquitinates Nrf2 in the cytoplasm, resulting in its rapid degradation by the ubiquitin proteasome system (Iso et al., 2016; Sekine et al., 2016). Oxidative stress causes Nrf2 to separate from Keap1 and transfer to the nucleus, where it induces the expression of antioxidant genes (Bellezza et al., 2018). NQO1 is an antioxidant enzyme that is activated by the target gene products of the Nrf2-Keap1-ARE pathway (Ross and Siegel, 2021). Several previous studies have shown that activation of the Keap1/Nrf2/NQO1 signaling pathway has a protective effect on renal oxidative damage in diabetic models (de Haan, 2011; Jin and Chen, 2022; Zheng et al., 2011). We hypothesized that the Keap1/Nrf2/NQO1 signaling pathway might serve as a potential therapeutic target for PET to delay DN progression. Our *in vitro* and *in vivo* studies revealed that hyperglycemia significantly increased Keap1 expression and inhibited Nrf2 and NQO1 expression, whereas PET treatment reversed these trends and improved oxidative stress and ferroptosis. With these experiments, we found that Keap1 expression was negatively correlated with the PET dose. Furthermore, the overexpression of Keap1 could offset these protective effects of PET. Therefore, our results suggested that PET activated the Keap1/Nrf2/NQO1 signaling pathway to play a protective role in DN by inhibiting Keap1 expression.

Mitochondrial dysfunction can exacerbate oxidative stress and ferroptosis (Chen et al., 2023). SOD is a key enzyme for free radical detoxification in cells and plays a crucial role in maintaining the redox balance of mitochondria, and Mn-SOD is expressed in mitochondria (Flynn and Melov, 2013). Our results suggested that PET increased SOD2 levels to restore antioxidant activity by inhibiting Keap1 expression and ultimately contributed to maintaining mitochondrial homeostasis. NQO1 has been shown to have superoxide reductase activity (Siegel et al., 2004; Zhu et al., 2007) and prevent lipid peroxidation (Ross and Siegel, 2004). Multiple studies have shown that ferroptosis can be inhibited by targeting NQO1 (Wang et al., 2023; Yu et al., 2023; Zhan et al., 2022). In this study, we found that only NQO1 translocated to the mitochondria in response to the oxidative stress-related Keap1/Nrf2/NQO1 signaling pathway in db/db mice and HG-induced HK2 cells. Thus, mitoNQO1 overexpression could reduce the oxidative stress in cells and mitochondria induced by high glucose and reduce ferroptosis at the same time. This confirmed that the protective effect of PET on DN was partly mediated through the upregulation of mitoNQO1 expression. To further verify whether PET was affected by the Keap1/mitoNQO1 signaling pathway, we overexpressed mitoNQO1 and observed that PET maintained its protective effect. We then overexpressed Keap1, and the benefits were blocked. Therefore, the current data confirmed that the protective effects of PET against diabetic kidney injury might occur through the Keap1/mitoNQO1 pathway.

Numerous *in vitro* experiments have convincingly demonstrated the antioxidant potential of anthocyanin derivatives and anthocyanin - rich foods (Bibi Sadeer et al., 2020) Anthocyanins are capable of penetrating cells and activating the Nrf2/HO1 pathway, thereby providing protection against antioxidant damage and enhancing the antioxidant defense mechanism (Jiang et al., 2020). Rahman et al. (Rahman et al., 2006). reported that delphinidin, isolated from blueberry extract, exhibited the highest antioxidant capacity among the tested anthocyanidins, followed by petunidin > malvidin = cyanidin > peonidin > pelargonidin. Despite the promising antioxidant properties of anthocyanins, their therapeutic application encounters certain challenges. Specifically, only a minuscule proportion (<2%) of the initially ingested anthocyanins can be detected in the bloodstream (Manach et al., 2005). This limited recovery is attributed to the poor stability and low bioavailability of these compounds within the gastrointestinal tract (Fang, 2014).Notably, recent advancements in nanotechnology offer potential solutions to these problems. Several studies have indicated that encapsulating anthocyanins in nanoformulations can significantly improve the stability and bioavailability of these phytochemicals (Rashwan et al., 2023; Salah et al., 2020). Rajan et al. (2019) by regarding the structure of PET, detailed investigations have revealed that its pKa value is the lowest, even lower than the pH of blood. As a result, PET can exist in a deprotonated form in the blood, which is conducive to its good oral activity. PET demonstrates strong biological activity as a nuclear receptor ligand. Moreover, it exhibits potent inhibitory activity as a GPCR and ion channel modulator. Additionally, PET shows remarkable inhibitory effects against enzymes, proteases, and kinases. Collectively, these characteristics make PET a promising candidate for potential antioxidant and oral drug applications. There are currently no trials on its use as a drug in the human body.

However, there were several limitations to our study. First, although we predicted the binding of PET to Keap1 and elucidated the negative correlation between Keap1 protein expression and PET dose, direct evidence of their binding to each other is lacking. Second, although Western blot analysis revealed that downregulation of Keap1 expression promoted the mitochondrial translocation of NQO1, the specific mechanism involved remains to be further explored. Finally, whether PET has similar effects on other kidney diseases remains to be determined.

In conclusion, we demonstrated the protective effects of PET on DN. Mechanistic studies revealed that PET effectively inhibited Keap1 and promoted the translocation of NQO1 to mitochondria. These effects reduced the oxidative stress and ferroptosis associated with DN. These data suggest that the Keap1/mitoNQO1 pathway might be a promising therapeutic target for DN. Inhibiting Keap1 and increasing mitochondrial NQO1 levels might be beneficial for reducing diabetic kidney injury.

## Data Availability

The original contributions presented in the study are included in the article/Supplementary Material, further inquiries can be directed to the corresponding authors.
