# Supplementary material for: Petunidin alleviates diabetic nephropathy injury via the inhibition of oxidative stress and ferroptosis through the Keap1/mitoNQO1 pathway
Source: Front Cell Dev Biol. 2025 Oct 16;13:1651382. doi: 10.3389/fcell.2025.1651382 (PMC12574455; doi:10.3389/fcell.2025.1651382)
Supplement: Supplementary file 1 [file Table1.docx]

Supplementary Material

# Supplementary Table 1


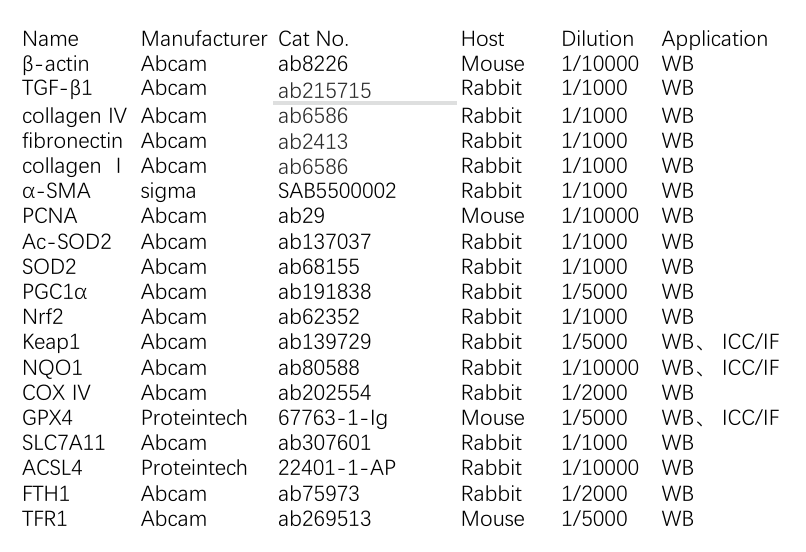


**Supplementary Table 1.** The primary antibodies used for Western blotting
